# Engystol reduces onset of experimental respiratory syncytial virus-induced respiratory inflammation in mice by modulating macrophage phagocytic capacity

**DOI:** 10.1371/journal.pone.0195822

**Published:** 2018-04-19

**Authors:** Sabine Wronski, Julia Dannenmaier, Sabine Schild, Olaf Macke, Laura Müller, Yvonne Burmeister, Bernd Seilheimer, Meike Müller

**Affiliations:** 1 Department of Preclinical Pharmacology and Immunology, Fraunhofer Institute for Toxicology and Experimental Medicine, Hannover, Germany, Biomedical Research in Endstage and Obstructive Lung Disease Hannover (BREATH), Member of the German Center for Lung Research; 2 Biologische Heilmittel Heel GmbH, Baden-Baden, Germany; Washington State University, UNITED STATES

## Abstract

**Background:**

Respiratory viruses such as respiratory syncytial virus (RSV) or rhinovirus are one of the major causes for respiratory tract infections causing common cold disease. Respiratory viral infections range from mild symptoms in adults to serious illness especially in the very young or elderly as well as patients suffering from lung diseases or being immunocompromised due to other reasons. Engystol (EGY-2) is a multicomponent, multitarget preparation consisting of Vincetoxicum hirundinaria and Sulfur in various dilutions. The study objective was to test the effect of EGY-2 on the innate immune response during the early onset of respiratory viral infection in vivo as exemplified in a mouse model of RSV-induced respiratory inflammation.

**Methods:**

Naïve BALB/c mice were infected with 1x10^6^ infectious units RSV A2 intranasally to cause a mild respiratory infection. EGY-2 was administered daily per oral gavage starting seven days prior to RSV infection at doses of 0.4 to 5.1 tablets/kg. Control groups received placebo treatment. Animals were sacrificed 1 to 3 days post infection (p.i.) to analyse the infection and induced immune response in the lung. Viral load in bronchoalveolar lavage fluid (BALF) and lung homogenate was determined by TCID_50_ assay as well as immunofluorescence staining of BALF cells using anti-RSV antibody and microscopic analysis. The RSV induced immune response was assessed by evaluation of BALF differential cell count, BALF cytokine secretion and analysis of the phagocytic capacity of alveolar macrophages.

**Results:**

EGY-2 significantly reduced the RSV induced neutrophil and early lymphocyte influx on day 1 p.i. in BALF. EGY-2 treatment significantly diminished the RSV induced secretion of pro-inflammatory cytokines such as IFN-γ, IL-1β, IL-6, KC and TNF-α at day 1. EGY-2 treatment was not protective for RSV infection per se, as no alteration in the viral load in lung and BALF was detected. Enhanced numbers of phagocytic-active macrophages were observed in EGY-2 treated animals on day 1 and this macrophage population showed strongly enhanced phagocytic activity on day 1 and day 3.

**Conclusion:**

The data suggest a beneficial immunomodulatory effect of EGY-2 during early onset of respiratory viral infection in vivo, mediated by stimulation of macrophage phagocytosis, resulting in a reduced innate inflammatory response in terms of neutrophil and early lymphocyte infiltration as well as reduced inflammatory cytokine secretion.

## Introduction

The common cold is one of the leading causes for consultation of a physician. Despite its generally mild course of disease the common cold continues to be a massive burden in terms of quality of life and socioeconomic impact due to its high prevalence [1,2]. Furthermore, symptoms can be much more severe and even life-threatening in vulnerable populations like the very young or elderly as well as patients suffering from lung diseases like COPD or being immunocompromised e.g. due to immunosuppressive treatment after transplantations. The main causes of the common cold are respiratory viruses, such as rhinovirus or respiratory syncytial virus (RSV) [1–3]. While rather innocuous as common cold pathogen in adults, RSV can cause severe and life-threatening conditions such as pneumonia and bronchiolitis in infants with a high mortality rate [4]. Both rhinovirus and RSV are commonly used in in vitro and in vivo respiratory infection models for preclinical research and further investigation of the complex inflammatory mechanisms of the host-pathogen-interaction.

Upon entry into the respiratory system, airway epithelial cells (AEC) and alveolar macrophages (AM) represent the first site of encounter of the host with the invading pathogen. AM are the predominant immune cells in the airways and are important in regulating airway homeostasis [5]. In the normal host, and absence of infection, AM act as first line phagocytes responsible for clearance of any inhaled particulate matters. Importantly, AM and AEC are equipped with a variety of receptors, e.g. pattern recognition receptors (PRR) that enables them to act as guardians of the airways, raising alarm upon encounter of dangerous pathogens like RSV among the inhaled particulates [5,6]. Recognition of pathogen-associated molecular patterns (PAMP) by the PRR, or activation through cytokine mediators released from infected epithelial cells, leads to secretion of a variety of chemokines and cytokines alerting the host to induce an appropriate immune response.

Primarily infected AEC release a variety of chemokines such as CCL5 (RANTES), CCL2 (MCP-1), CXCL10 (IP-10), and CXCL8 (IL-8) [6,7]. These chemokines recruit and activate inflammatory cells especially monocytes/macrophages, T cells, NK cells and neutrophils. Despite epithelial cells being the primary target of infection, AM play the major role in inducing the protective host immune response [8]. AM as main producers of type I interferons [9] exhibit antiviral effects directly or by activating interferon response factors leading to inhibition of viral replication and enhanced viral clearance. AM are furthermore important producers of pro-inflammatory cytokines such as TNF-alpha, IL-6 and IL-8 [8,10], as well as the anti-inflammatory cytokine IL-10 [11] upon infection with respiratory viruses such as RSV.

At present only limited therapeutic options for RSV are available in the market. The only licensed curative drug ribavirin, a virostatic agent, has been shown to have limited efficacy and safety and is only suggested for high risk infants or immunocompromised patients [12].

As respiratory virus induced illness has been described to result predominantly from the inflammatory host response, with severity of the disease being linked to the inflammatory mediator production [13], a new strategy of treatment is the use of immunomodulatory compounds targeting the host cells rather than acting directly anti-viral [14]. As AM are key regulators of the airway immune response, their regulatory network represents a promising therapeutic target.

Engystol (EGY-2) is a multicomponent, multitarget homeopathic medicinal product frequently used for prophylactic and acute treatment of viral infections (e.g. common cold). EGY-2 is commonly prescribed during early stages of common cold, or even preventive during the cold season. The therapeutic effect of EGY-2 has been described in several studies. In an observational study EGY-2 was used for the treatment of upper respiratory infections associated with common cold and was non-inferior in comparison to over the counter therapies [15]. In a double-blind trial EGY-2 indicated a more rapid recovery from illness as compared to the placebo group [16]. Moreover, observational studies demonstrated that EGY-2 is well tolerated [15,17]. In vitro studies indicated that EGY-2 exhibits antiviral activity [18,19]. It was shown that EGY-2 possesses antiviral activity against a broad spectrum of viruses [18,19] which in certain cases (e.g. HSV-1 infection) may be exerted by increased type I IFN release [19]. However, the exact mode of action of EGY-2 is still under investigation. Within this study, a mouse model of mild RSV-induced respiratory tract infection served as a model to mimic common cold disease in order to study the effects of EGY-2 on the early onset of infection and its modulatory effects on the early innate immune response in vivo.

## Materials and methods

### Ethical statement

The study was conducted in accordance with the Regulations of the German Animal Protection Law (Tierschutzgesetz of May 18, 2006, BGBl. I S. 1206, 1313; adopted July, 28 2014 BGBl. I S. 1308) and European Council Directive on the protection of animals used for scientific purposes (2010/63/EU). The experiments were approved by the local authority (Lower Saxony Federal State Office for Consumer Protection and Food Safety, reference no. 33.14-42502-04-14/1428).

### Animals

Wild-type, female BALB/c mice (6–8 weeks old) were purchased from Charles River (Sulzfeld, Germany). All animals were held in regular 12 h dark/ light cycles at 22 ± 2°C and 55 ± 15% relative air humidity and received laboratory food and tap water ad libitum. Animals were acclimatized for two weeks before the experiments started. At start of the study animals were 8–10 weeks old with a body weight of approx. 18 ± 0.7 g. The health condition of the animals was monitored daily.

### Test items

The test item EGY-2 (marketed under the brand name Engystol) and the respective placebo tablets were supplied by Biologische Heilmittel Heel (Baden-Baden, Germany). EGY-2 contains two components in different dilutions: Vincetoxicum hirundinaria (D6), Vincetoxicum hirundinaria (D10), Vincetoxicum hirundinaria (D30), Sulfur (D4) and Sulfur (D10). The multicomponent test item was manufactured in Germany according to GMP standards. Working solutions were prepared freshly on every application day by dissolving tablets of EGY-2 or placebo in water and appropriate dilution to yield doses of 0.4, 2.57 or 5.1 tablets/kg in 200 μL volume.

### Virus

Respiratory syncytial virus (RSV) strain A2 was obtained from Virapur (San Diego, CA) as semi-purified stock. The virus solution was thawed and diluted in PBS to yield an inoculum of 1x10^6^ infectious units / 50 μL according to the titer given in the datasheet. The working solution was pretested in naïve BALB/c mice to select the optimal inoculum titer to induce infection in vivo as indicated by neutrophilic inflammation in the BALF 1 d p.i.. The UV-inactivated virus served as a control and confirmed the specificity of the induced response.

### Respiratory virus-induced pulmonary inflammation model and pharmacological treatment

Prior to the experiment, a total of 86 animals were weighed and randomly allocated to the treatment groups (n = 20/group, 10 animals per time point). After distribution in groups the standard deviation of mean body weights were checked to be below 20% within each group and between groups. The groups used in the study included three RSV infected EGY-2 treated groups, one infection control group (RSV infected, placebo-treated) and a negative sham infected, placebo-treated group (n = 6/group, 3 animals per time point). Infection was induced by intranasal inoculation of 1x10^6^ RSV in 50 μL volume per mouse under light inhalative isoflurane anesthesia. Control animals received PBS for sham infection. The animals were treated daily by oral gavage with EGY-2 at doses of 0.4, 2.57 or 5.1 tablets/kg in 200 μL starting on day -7 until the last day before necropsy which was performed on day 1 or 3 post infection (p.i.). Control groups received placebo (5.1 tablets/kg) instead.

As a control experiment, uninfected animals (n = 6 per group) were treated daily by oral gavage with EGY-2 at doses of 0.4, 2.57 or 5.1 tablets/kg in 200 μL over 8 days equal to the treatment schedule of the infection study and necropsy was performed 1 day after the last treatment.

### BALF and lung tissue collection

Animals were sacrificed painlessly with an overdose of pentobarbital sodium (Narcoren^®^) at 1 or 3 days after RSV infection. Bronchoalveolar lavage (BAL) was performed by intubating the lungs of the animals and lavaging two times, each time with 0.8 mL ice cold 0.9% NaCl. For measurement of the viral load, the right lung lobes were dissected after perfusion with ice cold PBS via the *atrium sinistrum*. The lung tissue was mechanically disintegrated with 0.5 mL ice cold PBS using fast-prep lysing matrix D tubes with an automated tissue homogenizer (Fast Prep®; MP Biomedicals). The cell suspension was centrifuged for 10 min at 300 x g and the supernatant was filtered through a 40 μm filter and viral load determined by TCID_50_ titer assay.

### Virus titer assay

The BALF supernatant and lung homogenates were analysed for virus load by titration on Hep-2 cells using two-fold serial dilution steps. After incubation at 37°C, 5% CO_2_ for 3 up to a maximum of 6 days, the CPE was assessed for each dilution to calculate the virus titer. The TCID_50_ (tissue culture infective dose 50) was calculated using the Spearman-Karber formula.

### BALF differential cell count and RSV staining

The BALF supernatant was aliquoted after sedimentation of the cells by centrifugation for 10 min at 300 x g at 4°C. BALF supernatant aliquots were frozen at -70°C. Cells were resuspended in 0.3 mL medium (RPMI without phenol red/ 20 mM Hepes) and counted automatically in a Casy® cell counter. Per animal two cytospots were prepared, one was stained according to Pappenheim to evaluate differential cell counts, counting a total number of 300 cells per cytospin. The second cytospin was used to assess the relative number of RSV-positive BALF cells. Therefore, BALF cytospins were fixed with acetone, stained with anti-RSV-FITC antibody (Merck, Darmstadt, Germany) for 30 min at 37°C in humid atmosphere and after several washing steps analysed for immunofluorescence (Zeiss Axioscope 2 plus, Zeiss, Jena, Germany).

### BALF cell phagocytosis assay

BALF cells were analysed for phagocytic activity by flow cytometry using pHrodo™ Red *E*. *coli* BioParticles® Conjugate (MolecularProbes/ Thermo Fisher Scientific, Schwerte, Germany). Therefore, BALF cells were resuspended in medium (RPMI without phenol red/ 20 mM Hepes) and adjusted to a cell count of 1x10^6^/mL. 100 μL (1x10^5^ cells) were incubated for 2 h in 5 mL tubes with pHrodo *E*.*coli* or medium at 37°C or 4°C as a negative control in a total volume of 200 μL medium. To minimize cell adhesion to tube walls polypropylene tubes were used and after incubation, cells were put on ice. For flow cytometric analysis 300 μL PBS were added per tube and cells were directly analysed for phagocytosis using a Beckman Coulter FC500 flow cytometer. Due to the limited cell number per tube, controls were pooled for each group.

### BALF cytokine analysis

The BALF supernatant samples were analysed using MSD pro-inflammatory panel V-Plex including the following analytes: IFN-γ, IL-10, IL-12p70, IL-1β, IL-2, IL-4, IL-5, IL-6, KC/GRO and TNF-α (Meso Scale Diagnostics, Rockville, MD). The analysis was performed according to the manufacturer’s instruction.

### Statistics

Biometric sample size calculation was performed to determine the group size for the study (Software: Statistica 9.1). Data in the figures are given as bar graphs or line plots with means + SD or box and whiskers plots with median and interquartile range or for normally or non-normally distributed data, respectively, as given in the figure legends. For analysis of significant differences between multiple groups statistical analysis was performed as follows: for normally or non-normally distributed data, ANOVA or Kruskal-Wallis test with parametric or non-parametric post hoc test, respectively, was used (Software: GraphPad Prism 4. version 4.03). Differences between treatment groups and controls were considered as statistically significant at the level of p < 0.05.

## Results

### EGY-2 reduced virus-induced neutrophil recruitment to BALF

**Fig 1:**
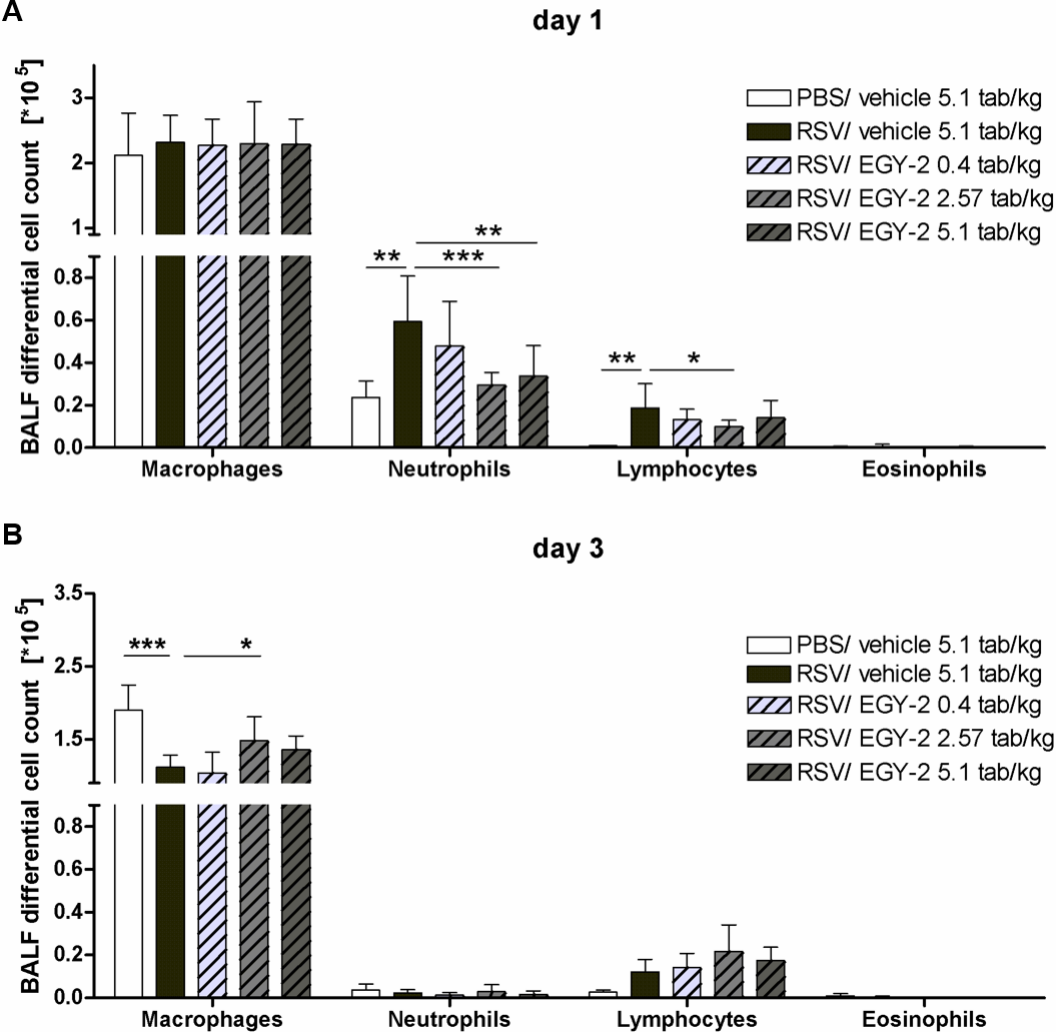
EGY-2 reduced virus-induced inflammatory cell infiltration to BALF. BALF absolute cell numbers [x 10^5^] for day 1 p.i. (A) and day 3 p.i. (B) are given as mean + SD for n = 10 animals per group (3 for negative control). *;**;*** indicates significance with p < 0.05; 0.01; 0.001 (One-way ANOVA and Bonferroni post test).

In the mild model of virus induced common cold used for the study, RSV infection, induced an inflammatory immune response characterized by early infiltration of predominantly neutrophilic granulocytes as indicated by the significant increase of BALF neutrophil numbers on day 1 p.i. Furthermore, lymphocyte cell counts in BALF were significantly higher on day 1 and day 3 p.i. compared to non-infected animals. At these early time points, reflecting the onset of disease, the inflammatory cell infiltration was efficiently decreased by EGY-2 as neutrophil numbers in BALF were significantly reduced in the EGY-2 high and medium dose groups on day 1 p.i. (Fig 1A). Lymphocyte cell counts on day 1 were also reduced by EGY-2 treatment, with significant reduction observed in the medium dose group (Fig 1A). Interestingly, this was associated with a trend towards enhanced lymphocyte numbers in this dose group on day 3 p.i. (Fig 1B), which however did not reach statistical significance. On day 3 p.i., the absolute number of macrophages was reduced in RSV infected animals and was significantly reversed in the EGY-2 medium dose group (Fig 1B).

In a control experiment using uninfected mice, animals treated with EGY-2 in a similar treatment schedule as in the infected mice showed that EGY-2 has no effect on the BALF differential cell counts under these conditions (S1 Fig).

Histologic analysis of lung tissue confirmed the mild nature of the chosen virus infection model, as no prominent pathophysiological alterations and no strong inflammatory response were observed. HE-stained lung thin-sections showed no qualitative differences in cellular composition and tissue integrity in RSV-infected animals compared to healthy control mice (S2 Fig), supporting the mild nature of the applied RSV model as a mild common cold model. EGY-2 treated groups accordingly showed no alterations compared to the control groups, demonstrating that no adverse effects were observed on tissue level (S2 Fig).

### EGY-2 reduced virus-induced cytokines

**Fig 2:**
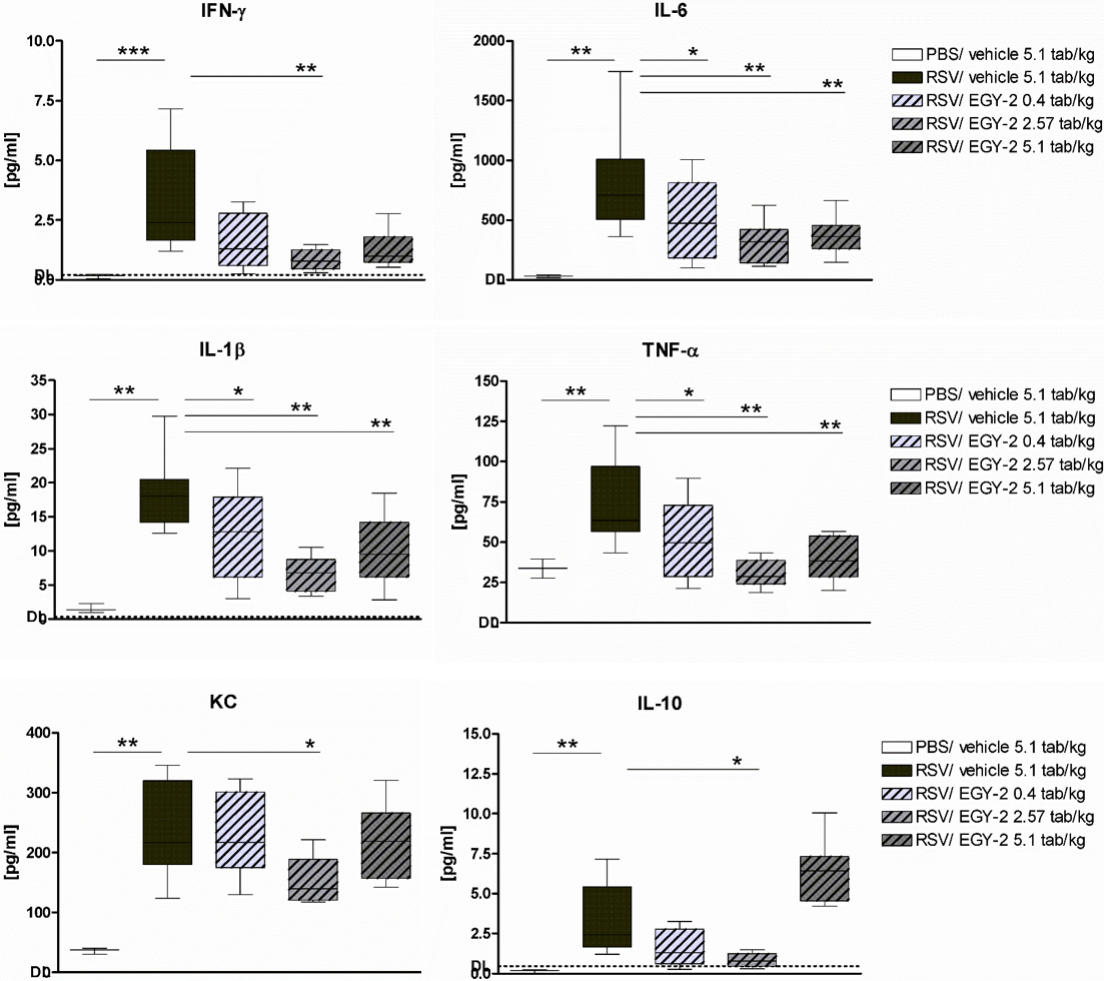
EGY-2 reduced virus-induced cytokine secretion. Cytokine levels [pg/mL] in BALF supernatant are shown for n = 10 animals per group (3 for negative control). Box-and-whiskers plots show median and interquartile range (box) and minimum/ maximum values (whiskers). *;**;*** indicates significance with p < 0.05; 0.01; 0.001 according to multiple testing using Kruskal Wallis and Dunn’s post test (IFN-γ, IL-10) or One-way ANOVA and Dunnett’s post test (IL-1β, IL-6, KC, TNF-α) as non-normally or normally distributed data, respectively. Lower detection limits are indicated as dotted line.

RSV infection induced secretion of pro-inflammatory cytokines IFN-γ, IL-6, IL-1β, TNF-α, KC as well as anti-inflammatory IL-10 measured in BALF day 1 p.i. (Fig 2). This early acute host response declined toward day 3 p.i. with cytokine levels comparable to uninfected animals (data not shown). At day 1 p.i. EGY-2 efficiently reduced this RSV induced release of pro-inflammatory cytokines, as BALF levels of IL-6, IL-1β and TNF-α were significantly reduced already at the lowest dose of 0.4 tab/kg and even more pronounced in the medium and high dose groups. IFN-γ was significantly reduced in the medium dose group, but also a trend to reduced levels was observed in the low and high dose group. For the neutrophil-chemoattractant KC, the mouse equivalent for IL-8, only in the medium dose group a significant reduction compared to sham treated control group was observed. For the anti-inflammatory cytokine IL-10, which is induced in parallel to the pro-inflammatory cytokines and acts to limit the inflammatory response, slightly reduced levels were observed in the low dose group and significantly reduced levels were observed in the medium dose group. However, in the high dose group this was not maintained and even a trend towards higher levels compared to control group was observed.

### EGY-2 effects on viral load

**Fig 3:**
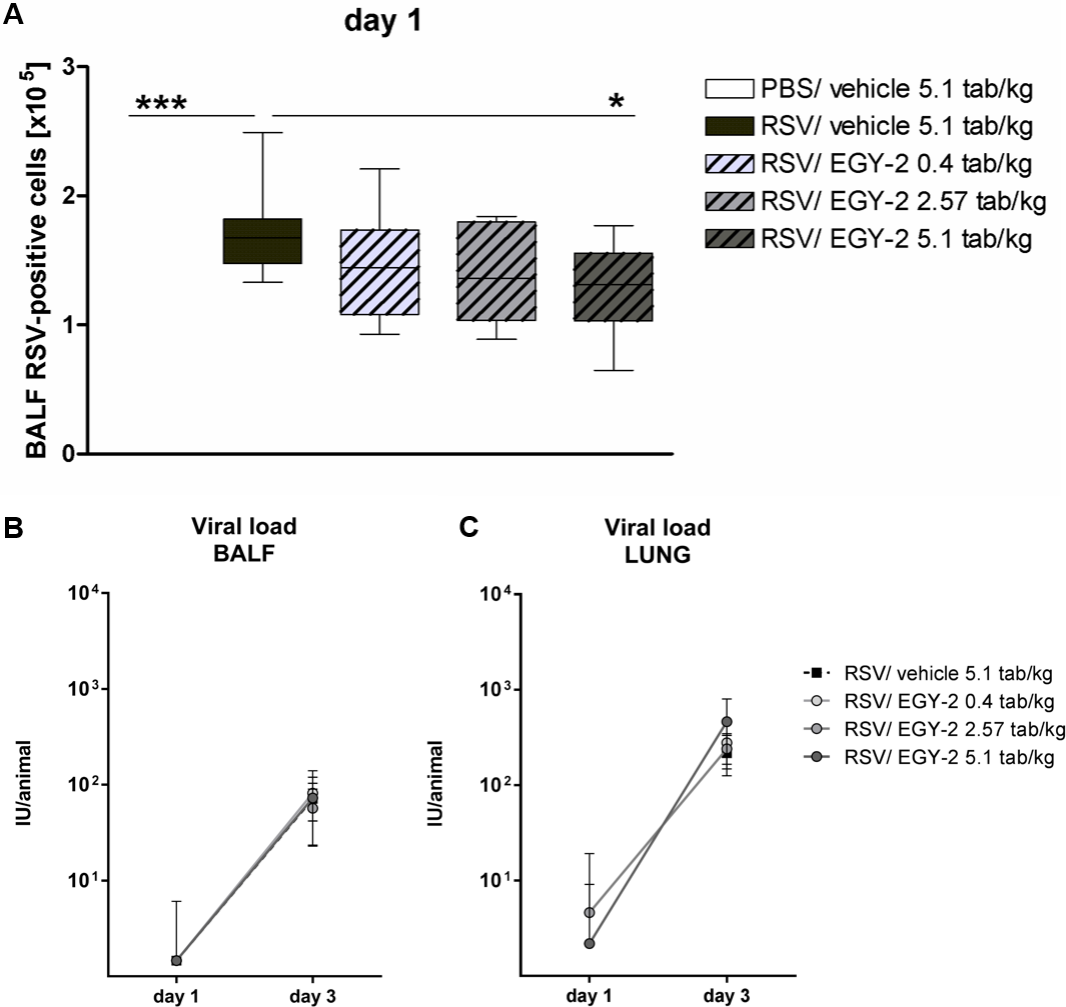
EGY-2 had no direct effect on viral load in BALF and lung tissue. Number of RSV-positive BALF cells (A) and viral load in BALF (B) and lung tissue (C) are shown for n = 10 animals per group (3 for negative control). (A) Box-and-whiskers plot shows absolute numbers x10^5^ as median, 25th to 75th percentile and maximum/minimum values. *;*** indicates significance with p < 0.05; 0.001 (One-way ANOVA and Bonferroni post test). (B+C) Results are shown as means ± SD.

The immunofluorescence staining of BALF cells with anti-RSV-FITC antibody, detecting mainly macrophages as infected epithelial cells cannot be assessed in lavage fluid, showed that approximately 50% of BALF cells of infected animals were RSV positive on day 1 p.i., thereby confirming presence of virus and phagocytosis by macrophages in vivo. EGY-2 in the highest dose of 5.1 tab/kg induced a slight but statistically significant reduction of RSV-positive BALF cells (Fig 3A). On day 3, only a small proportion (< 2%) of RSV-positive BALF cells was detected in all infected animals with no differences between groups (data not shown). Only a small proportion of replicating free virions could be detected by TCID_50_ assay in BALF and lung tissue on day 1 p.i., which subsequently increased towards day 3 p.i., confirming productive viral replication in vivo (Fig 3B and 3C). However, the detectable viral load in BALF and lung tissue was low and did not differ between treatment groups.

### EGY-2 increased number and activity of phagocytic alveolar macrophages

**Fig 4:**
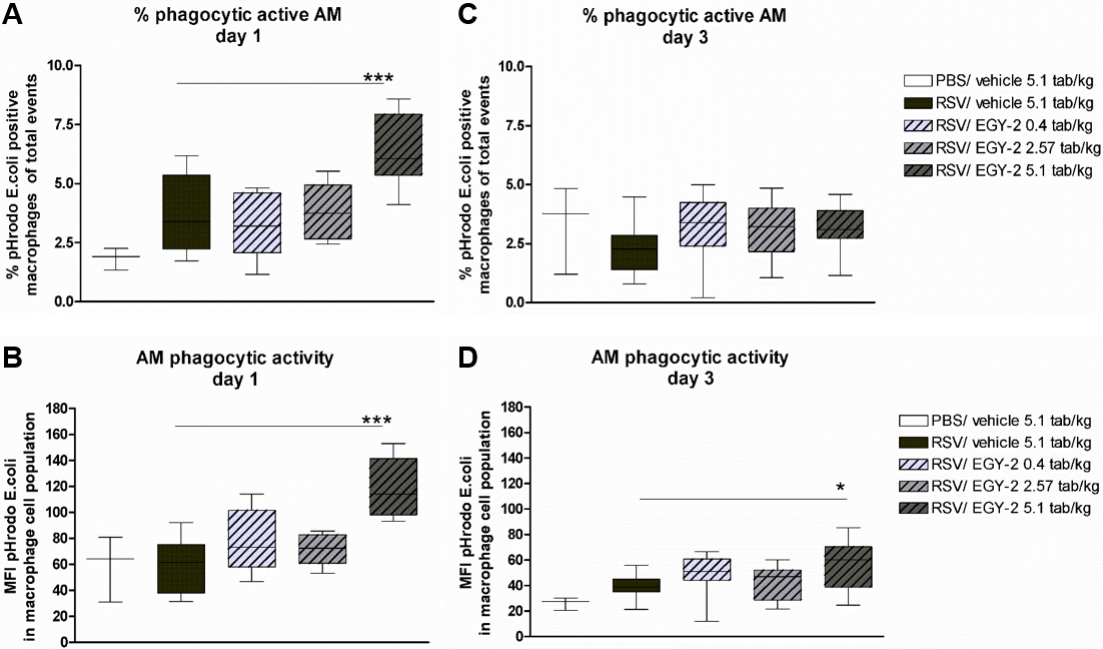
EGY-2 increased number and activity of phagocytic alveolar macrophages. Percentage of pHrodo-E.coli positive alveolar macrophages (AM) on day 1 p.i. (A) and day 3 p.i. (C) as well as phagocytic activity of these macrophages measured as mean fluorescence intensity (MFI) for pHrodo E.coli fluorescence on day 1 p.i. (B) and day 3 p.i. (D) is shown for n = 10 animals per group (3 for negative control). *; *** indicates significance with p < 0.05; 0.001 (One-way ANOVA and Bonferroni post test).

In RSV infected animals treated with EGY-2, alveolar macrophages (AM) in the high dose group showed a markedly enhanced phagocytic function at the early phase of infection, as both the number of phagocytic AM as well as their phagocytic activity was significantly enhanced compared to untreated animals on day 1 p.i. (Fig 4A and 4B, respectively). On day 3 p.i., the percentage of phagocytic AM decreased to basal levels (Fig 4C) potentially due to the previous activation upon RSV infection. However, the increased phagocytic activity in EGY-2 high dose group was maintained also on day 3 p.i. (Fig 4D).

In the control experiment using uninfected mice, EGY-2 did not show this stimulatory effect on macrophage phagocytosis (data not shown).

## Discussion

The purpose of this study was to evaluate the effects of the multicomponent multitarget immunomodulatory medicinal product EGY-2 on the innate immune response during early onset phase of virus-induced mild respiratory inflammation reflecting common cold. This was addressed using a mouse model of mild pulmonary inflammation induced by RSV as a common causative pathogen, investigating the early onset phase of disease up to d3 post infection. In the applied model, RSV infection induced an inflammatory host response, characterized by a predominant influx of neutrophils. Furthermore, early lymphocyte infiltration was observed on day 1 p.i., which presumably reflects an increase in natural killer (NK) cells, as these cells are phenotypically indifferent from classical lymphocytes in the BALF differential cell count assay. NK cells are known to be present in the lung, increase early during RSV infection and are an important source of IFN-γ [20,21]. In the present study increased IFN-γ levels were confirmed in the BALF of RSV-infected animals. IFN-γ, produced by NK cells and T cells, has a protective role by acting directly antiviral via inhibition of viral replication, but also activates macrophages. [22].

EGY-2 treated animals showed significantly reduced numbers of neutrophil and early lymphocyte infiltration accompanied by significant reduction of pro-inflammatory cytokines such as key macrophage cytokines TNF-α, IL-6 and IL-1β released upon RSV infection. EGY-2 furthermore significantly reduced levels of IFN-γ in the BALF, which was in line with the reduced early lymphocyte influx suggesting a reduced NK cell activation.

The increased amount of KC (IL-8) as key chemoattractant for neutrophils reflects the activation of epithelial cells upon viral infection in line with the macrophage activation. In the EGY-2 medium dose group, the KC level was significantly reduced. This strongly corresponds to the neutrophil cell counts in BALF that were similarly affected by EGY-2.

Interestingly, BALF levels of the anti-inflammatory cytokine IL-10 was reduced in EGY-2 medium dose group, while a contrary trend towards higher levels was observed in the EGY-2 high dose group. This suggests that in the present study at lower doses of EGY-2, IL-10 might be reduced similar to the pro-inflammatory cytokines like IFN-γ, IL-6, IL-1β and TNF-α due to the overall dampened or potentially earlier occurring inflammatory immune response, while at the high dose EGY-2 might additionally support the anti-inflammatory response by inducing IL-10. Studies in IL-10 -/- mice infected with RSV have shown a more severe disease phenotype compared to wild type mice, highlighting the important regulatory function of IL-10 during RSV induced disease [23]. Whether the induction of IL-10 by high doses of EGY-2 relates to an aspect of the potential mode of action, leading to the diminished virus-induced pathophysiological immune response needs further investigations.

The dampened inflammatory immune response towards respiratory virus infection in EGY-2 treated animals was linked to enhanced phagocytic capacity of alveolar macrophages. Phagocytosis by AM as first line defence is the initial step to induce the innate immune response of the host. In general, during RSV infection, the numbers of phagocytic active AM decreases towards later time points, potentially reflecting some kind of “exhaustion” of the macrophage response. Furthermore, RSV has been described to interfere with macrophage phagocytosis [24]. EGY-2 treatment was able to enhance the AM phagocytic activity even at day 3 p.i., which indicates that EGY-2 is able to support the AM defensive function even in the “exhaustion” phase.

Interestingly, we observed that EGY-2 showed the same stimulating effect on AM phagocytosis in mice infected with an RSV inoculum exhibiting lower infectivity thereby not inducing a neutrophilic driven inflammatory response but still showing productive viral replication in vivo (data not shown). Even under this condition of virus presence without active immune response, the percentage and activity of phagocytic macrophages was significantly enhanced by EGY-2 and persisted until day 3 p.i., indicating that EGY-2 supports the AM phagocytosis also in a non-inflammatory environment (data not shown). This might be interpreted to be in line with the order of engagement during immune responses: in order to restrict immunopathology (and higher collateral damage) as a result of excessive immune response [25]. In this case, this would mean that neutrophils are recruited only if tissue-resident macrophages alone cannot restrict the infection. Thus, the beneficial effects of EGY-2 on the RSV-induced immune response might be achieved by supporting macrophage functions inducing an improved immune response and limiting neutrophil recruitment which might subsequently lead to an earlier resolution of the acute inflammatory response.

In the present study, the EGY-2 effect on macrophage phagocytosis was not directly reflected as an impact on the presence of RSV in the respiratory compartment, as neither BAL fluid nor lung tissue showed altered viral loads in the EGY-2 treated animals. The viral titer that could be detected after re-isolation from BALF and lung tissue was low compared to the inoculum, which can be attributed mainly to significant clearance during the first day of infection by innate immune cells. Similar viral titer patterns during RSV infection have been described before [26]. Despite this, the small proportion of active virus increased towards day 3, demonstrating active viral replication in vivo. However, in the present study only small proportions of virus could be detected by the TCID_50_ assay. Thus, clearing mechanisms present in vivo might mask direct anti-viral effects of EGY-2, which were previously reported in an in-vitro study [18]. Another in vitro study did not show any direct anti-viral effects versus RSV [19], which might be due to different treatment regimens. Interestingly, in the present study a slight but significant reduction of RSV-positive BALF cells was observed in EGY-2 treated animals on day 1 p.i. RSV-positive BALF cells mainly reflect macrophages, as epithelial cells are not accessible by lavage. In contrast to epithelial cells however macrophages are thought to be not productively infected in terms of intracellular viral replication as appears in epithelial cells [27], but rather are detected positive due to their phagocytic uptake of RSV. Thus in light of the increased phagocytic activity of the AM, a reduction of RSV signal within macrophages might hint towards improved viral clearance via more rapid phagocytosis and destruction of the pathogen. The minimal amount of viral load detectable on day 1 p.i. in BALF and lung tissue in all groups though does not allow further conclusion if the viral clearance at earlier time points was improved in EGY-2 treated groups and needs to be addressed in future studies.

## Conclusions

In summary, the data indicate a potent immunomodulatory function of EGY-2 beneficial during inflammatory conditions in the lung, e.g. during the early onset of respiratory virus infection as shown in the present in vivo study. The multicomponent multitarget preparation is able to modulate macrophage function and their decisive role in initiating an immune response in order to further maintain airway homeostasis. EGY-2 supports macrophage function in the innate antiviral defence, improving their phagocytic activity while dampening the release of RSV-induced inflammatory cytokines. This results in a diminished inflammatory reaction of the host during the early onset of infection. If the observed effects of EGY-2 finally lead to an altered disease progression remains to be shown in future studies focusing on the outcome of the disease, which was beyond the scope of this study. However, the beneficial effects of EGY-2 exhibited during onset of the disease could pave the way for limiting the pathophysiological progress and improved resolution of the disease, as was seen in the observational studies.

## Supporting information

S1 Fig EGY-2 does not alter cellular composition of BALF in uninfected mice. BALF absolute cell numbers [x 10^5^] for day 1 after last treatment are given as mean + SD for n = 6 animals per group.
(TIF)

S2 Fig Histologic analysis of lung tissue–day 1 post infection. Representative photomicrographs (3 animals of each group) of H&E stained lung sections of mice after RSV (B-E) or sham infection (A) and treatment with EGY-2 in the indicated doses (C-D) or vehicle (A-B). Original magnification x25. Scale bar = 100μm.
(TIF)

S3 Fig Histologic analysis of lung tissue–day 3 post infection. Representative photomicrographs (3 animals of each group) of H&E stained lung sections of mice after RSV (B-E) or sham infection (A) and treatment with EGY-2 in the indicated doses (C-D) or vehicle (A-B). Original magnification x25. Scale bar = 100μm.
(TIF)

## Abbreviations

AEC: airway epithelial cells
AM: alveolar macrophages
BAL(F): bronchoalveolar lavage (fluid)
CPE: cytopathic effect
EGY-2: Engystol
IFN: interferon
IL: interleukin
KC/ GRO: Keratinocyte chemoattractant/ human growth-regulated oncogene
NK: natural killer (cells)
PAMP: pathogen associated molecular patterns
PBS: phosphate-buffered saline
p.i: post infection
PRR: pattern recognition receptors
RSV: respiratory syncytial virus
TCID_50_: tissue culture infective dose 50
TNF: tumor necrosis factor
