# Supplementary figures and images for: Engystol reduces onset of experimental respiratory syncytial virus-induced respiratory inflammation in mice by modulating macrophage phagocytic capacity
Source: PLoS One. 2018 Apr 19;13(4):e0195822. doi: 10.1371/journal.pone.0195822 (PMC5909611; doi:10.1371/journal.pone.0195822)

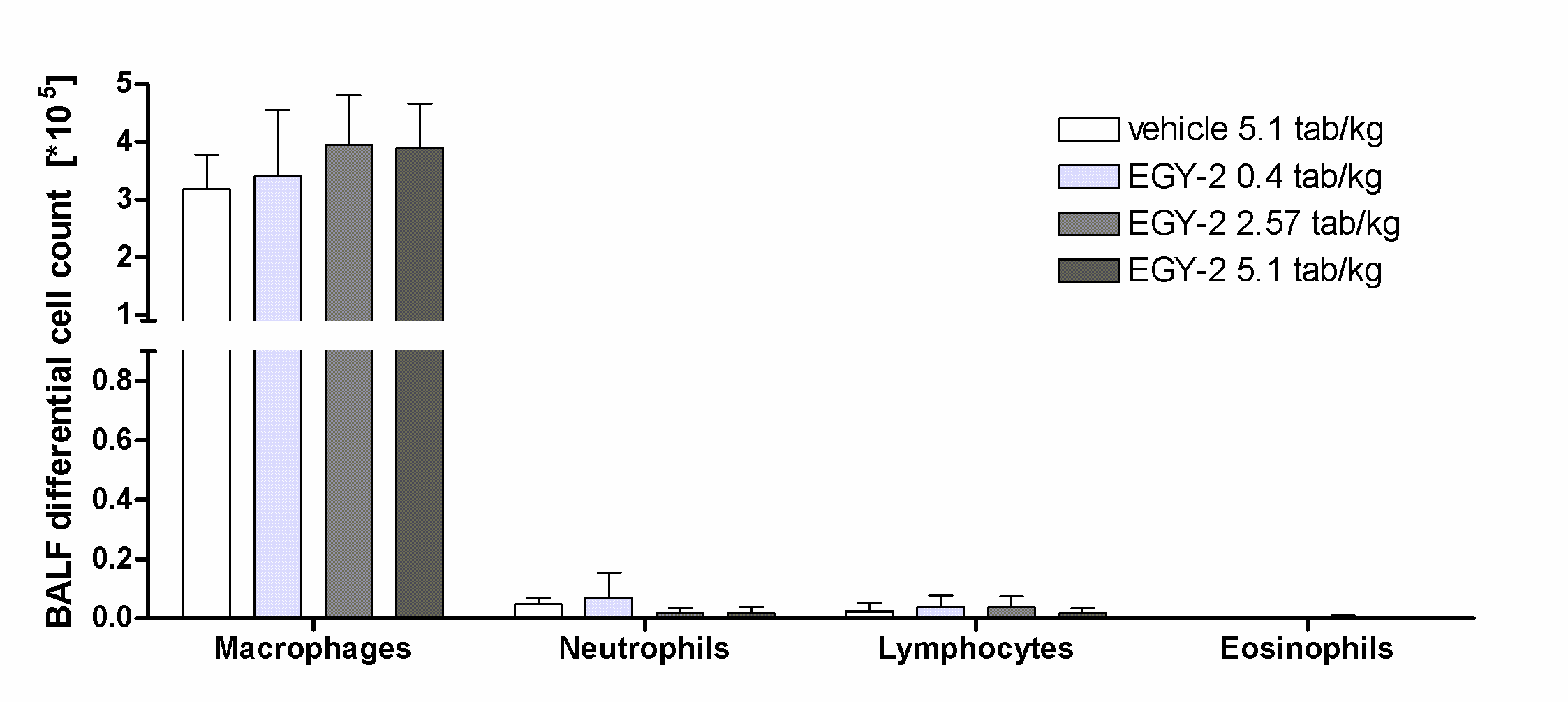

Supplement: S1 Fig — BALF absolute cell numbers [x 105] for day 1 after last treatment are given as mean + SD for n = 6 animals per group. (TIF) [file pone.0195822.s001.tif]

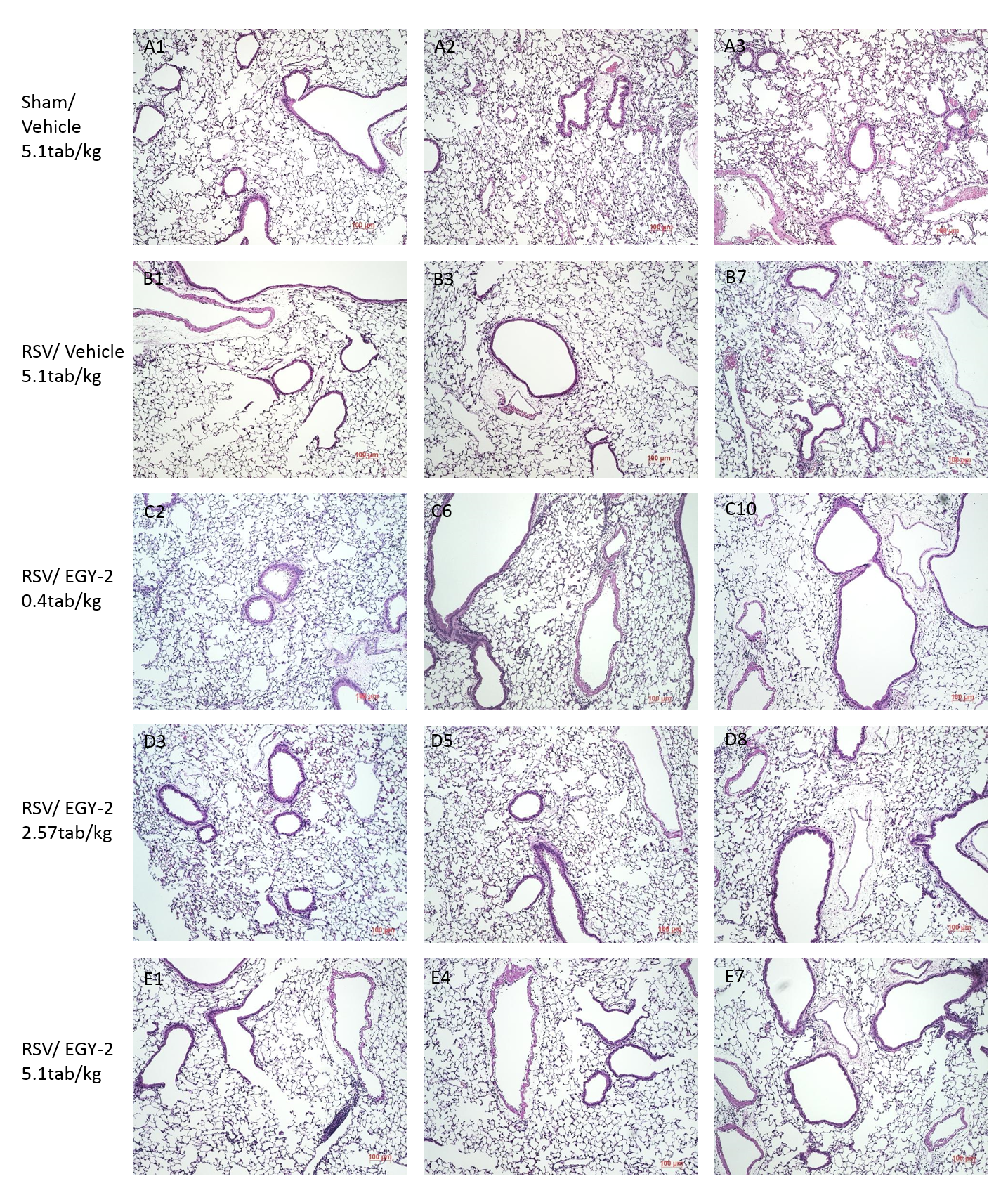

Supplement: S2 Fig — Representative photomicrographs (3 animals of each group) of H&E stained lung sections of mice after RSV (B-E) or sham infection (A) and treatment with EGY-2 in the indicated doses (C-D) or vehicle (A-B). Original magnification x25. Scale bar = 100μm. (TIF) [file pone.0195822.s002.tif]

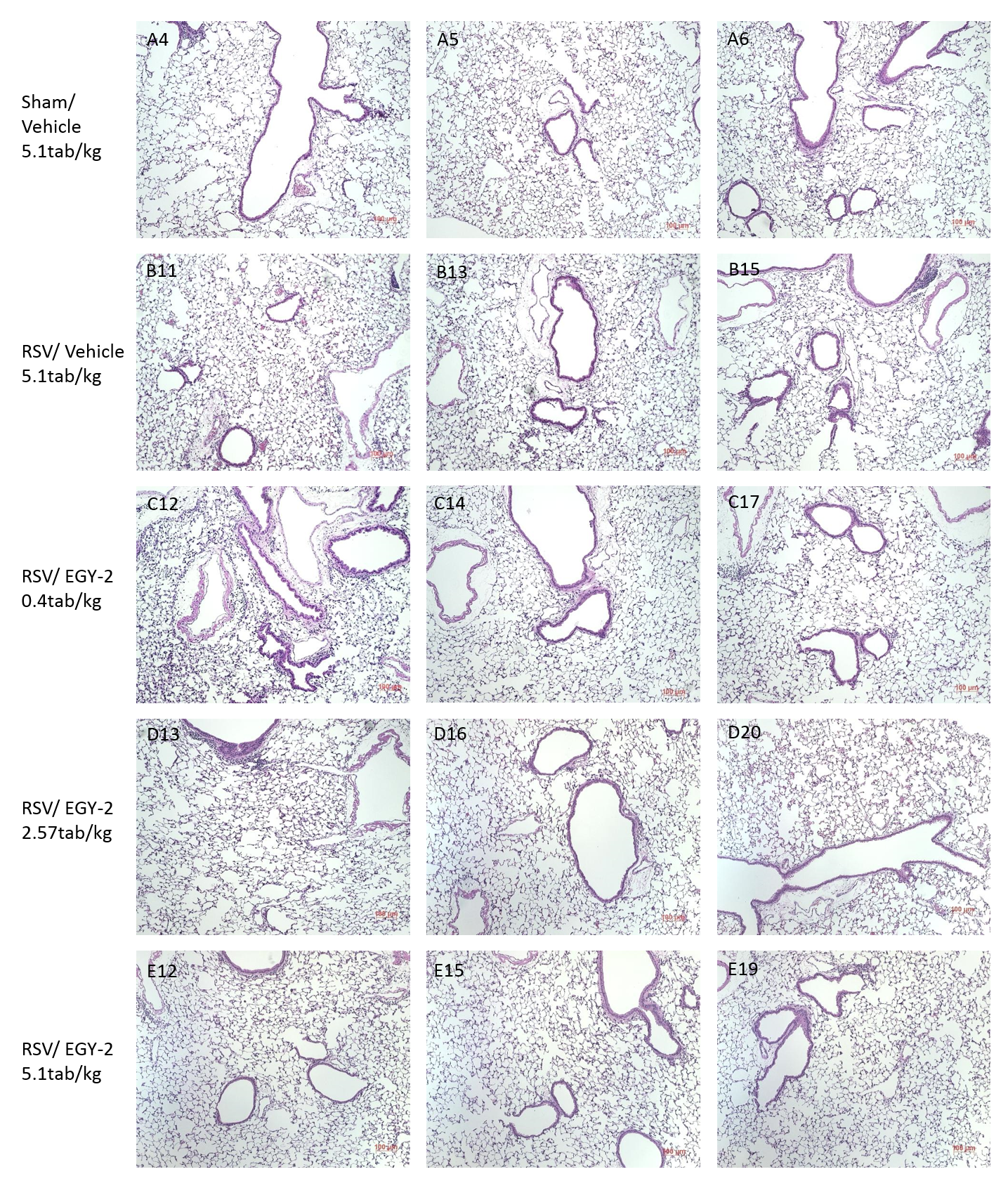

Supplement: S3 Fig — Representative photomicrographs (3 animals of each group) of H&E stained lung sections of mice after RSV (B-E) or sham infection (A) and treatment with EGY-2 in the indicated doses (C-D) or vehicle (A-B). Original magnification x25. Scale bar = 100μm. (TIF) [file pone.0195822.s003.tif]
